# Chronic Illness Burden Regular Healthcare Providers and Telehealth Use among African American and Latino Public Housing Residents

**DOI:** 10.21203/rs.3.rs-11537261/v1

**Published:** 2026-10-29

**Authors:** Mohsen Bazargan, Ehsan Yaghmaei, Attallah Dillard, Shervin Assari

**Affiliations:** Charles R. Drew University of Medicine and Science

**Keywords:** telehealth, racial and ethnic minorities, public housing, intersectionality, health disparities, mediation analysis, health needs, chronic illness

## Abstract

**Background:**

Access to an established healthcare provider may help explain differences in telehealth use among residents of public housing.

**Objective:**

As a continuation of Cobb et al. (2025), this study used the same survey dataset to examine whether having a regular healthcare provider may account for the association between chronic illness burden and telehealth use.

**Methods:**

This cross-sectional analysis included 252 African American and Latino adults living in four public housing developments in South Los Angeles. A structural equation model examined associations among chronic illness burden, regular provider status, and telehealth use, accounting for age, gender, education, and language.

**Results:**

Chronic illness burden was positively associated with having a regular provider (B = 0.158, p = 0.015), and regular provider status was positively associated with telehealth use (B = 0.145, p = 0.016). The adjusted direct association between chronic illness burden and telehealth use was not statistically significant (B = 0.094, p = 0.141).

**Conclusion:**

The observed associations are consistent with a possible pathway through access to a regular provider. They do not establish mediation or causal direction. Improving access to ongoing care may be relevant to efforts to support telehealth use in this population.

## 1. Background

Telehealth use among public housing residents needs to be understood within the conditions in which care is sought. Housing disadvantage and racial inequities may shape access to both local healthcare and the resources needed for remote visits [1-6]. An intersectional approach considers how these conditions occur together, rather than treating income, racial and ethnic identity, and housing as separate influences [7, 8]. For African American and Latino residents with limited financial resources, the availability of a clinician may be as relevant to telehealth use as the availability of technology.

A greater need for care does not necessarily translate into greater use of services. People with low incomes may encounter costs, transportation problems, and limited access to providers even when they have ongoing health concerns [9-13]. Residential segregation and housing policies may further shape where services are located and which communities can reach them [14-18]. Research on healthcare needs and service access in underserved settings supports examining the connection between illness burden and access to a regular source of care [19, 20].

Language and access to health information may also influence how residents seek care. Studies of Latino help-seeking and of health information use among underserved populations suggest that needs, preferences, and available resources differ across groups [23-28]. These differences make it useful to examine telehealth alongside established patterns of healthcare access, rather than assuming that a remote visit is equally accessible to all residents.

Cobb et al. [32] previously analyzed telehealth use in this same sample of African American and Latino public housing residents. That study examined perceived benefits and self-efficacy as potential mediators and did not find evidence supporting those pathways. The present analysis continues that work by shifting attention to an aspect of healthcare access: whether residents have a regular healthcare provider. Telehealth may reduce travel demands [29], but arranging a remote visit may still depend on having a clinical connection through which care can be obtained.

Using the same survey dataset as Cobb et al. [32], we examined whether chronic illness burden was associated with regular provider status and whether regular provider status was associated with telehealth use. The proposed pathway differs from the attitude-based pathways assessed previously. This is a secondary analysis of an existing sample, not an independent replication or a new round of data collection.

## 2. Methods

### 2.1 Study Design

This cross-sectional secondary analysis continues the research reported by Cobb et al. [32]. Both reports use the same face-to-face survey of public housing residents. The earlier publication focused on telehealth attitudes; the present analysis examines a proposed pathway from chronic illness burden through regular provider status to telehealth use.

### 2.2 Participants

The analytic sample comprised 252 adults who identified as African American or Latino and lived in four public housing developments in South Los Angeles. Interviews were conducted from August 2023 through January 2024. The four developments were located in Service Planning Area 6 and contained 2,306 housing units in total. They were selected from the 14 developments managed by the Housing Authority of the City of Los Angeles. Additional details about the setting and recruitment are available in Cobb et al. [32].

### 2.3 Sampling

Recruitment was nonrandom. Eligible residents were at least 18 years old, lived in Service Planning Area 6, identified as African American or Latino, and could complete an interview in English or Spanish. The current report uses the participants included in the earlier analysis [32].

### 2.4 Ethics

Participants gave written informed consent before the interviews. Charles R. Drew University of Medicine and Science approved the study protocol through its Institutional Review Board. The survey covered social and demographic characteristics, health conditions, and experiences with healthcare access and use [32].

### 2.5 Measurement

Measures were drawn from the survey described by Cobb et al. [32]. The present analysis retained the variables relevant to illness burden, regular provider access, and telehealth use.

#### 2.5.1 Socioeconomic and Demographic Variables

##### Age:

Age in years was reported by participants; observed ages ranged from 18 to 77.

##### Gender:

The survey recorded self-reported gender as female (1) or male (2).

##### Education:

Respondents selected their highest completed level of schooling, from eighth grade or less through a bachelor’s degree or higher. Table 1 combines the upper education categories, as in the earlier report [32].

##### Primary language:

Interview data distinguished English (1) from Spanish (2) as the participant’s primary language.

##### Chronic illness burden:

A count summarized participants’ self-reported diagnosed chronic conditions [32].

##### Regular healthcare provider:

Respondents indicated whether they had a doctor or other provider for primary care. Responses were coded no (0) and yes (1). This indicator represents access to a regular provider; it does not directly measure the duration or quality of continuity of care.

#### 2.5.2 Telehealth Utilization

##### Telehealth use:

The survey asked about healthcare visits conducted remotely using a telephone, smartphone, computer, or tablet [32].

##### Telehealth frequency:

Respondents reported the number of telehealth visits in the preceding 12 months. Table 1 presents the distribution of those visits.

### 2.6 Statistical analysis

Descriptive statistics and the relevant subset of bivariate correlations summarize the shared dataset previously reported by Cobb et al. [32]. The present structural equation model specifies chronic illness burden as a predictor of regular provider status and telehealth use, with regular provider status also predicting telehealth use. Age, gender, education, and primary language are included in both equations. Table 3 reports coefficients (B), standard errors, 95% confidence intervals, and p values.

## 3. Results

Table 1 describes the same 252 participants reported by Cobb et al. [32]. Their mean age was 45 years (SD = 15), and 188 (75%) were women. Educational attainment was eighth grade or less for 99 participants (39%), some high school without a diploma for 56 (22%), and high school completion or a GED for 66 (26%). The remaining 31 participants (12%) were in the combined category of some college, an associate degree, or a bachelor’s degree.

A regular provider was reported by 207 participants (82%), and 187 (74%) identified Spanish as their primary language. The chronic illness count was zero for 122 participants (48%), one for 47 (19%), two for 39 (15%), and three or more for 44 (18%).

During the preceding year, 133 participants (53%) reported no telehealth visits. The counts for one, two, three, and four or more visits were 50, 37, 19, and 13, respectively. These descriptive results overlap with the earlier publication because both analyses use the same sample [32].

Table 2 presents the subset of previously reported correlations relevant to this analysis [32]. Telehealth use was positively correlated with chronic illness burden and regular provider status (both r = 0.20, p < 0.01, at the precision shown in the table). Negative correlations with male gender (r = − 0.14) and Spanish language (r = − 0.15) were significant at p < 0.05. Age and education were not significantly correlated with telehealth use. These coefficients describe the shared dataset and are not new estimates from an independent sample.

Table 3 reports the model examined in this continuation study. Unlike the earlier model of telehealth attitudes [32], it includes regular provider status as an intermediate variable between illness burden and telehealth use. The reported coefficients are described below.

### Predictors of Telehealth Utilization

The analysis examined factors associated with telehealth utilization among low-income racial and ethnic minority residents of public housing in Los Angeles. Having a regular healthcare provider was positively associated with telehealth use (B = 0.145, SE = 0.060, 95% CI = 0.027, 0.264, p = 0.016), indicating that individuals with a designated provider were more likely to use telehealth services.

Gender was significantly associated with telehealth use (B = −0.153, SE = 0.060, 95% CI = −0.270, −0.035, p = 0.011), suggesting that females were more likely to engage with telehealth services compared to males. Additionally, Spanish as the primary language was negatively associated with telehealth utilization (B = −0.230, SE = 0.069, 95% CI = −0.365, −0.095, p = 0.001), implying that Spanish-speaking participants were less likely to use telehealth compared to English-speaking individuals.

Neither age (B = 0.049, SE = 0.073, 95% CI = −0.093, 0.192, p = 0.497) nor education level (B = −0.049, SE = 0.076, 95% CI = −0.197, 0.099, p = 0.517) showed a significant relationship with telehealth use. Similarly, the number of chronic illnesses was not significantly associated with telehealth utilization (B = 0.094, SE = 0.064, 95% CI = −0.031, 0.220, p = 0.141).

### Predictors of Having a Regular Healthcare Provider

The second model assessed predictors of having a regular healthcare provider, which was hypothesized to mediate the relationship between chronic illness and telehealth use.

Chronic illness burden was positively associated with having a regular healthcare provider (B = 0.158, SE = 0.065, 95% CI = 0.031, 0.285, p = 0.015), suggesting that individuals with a higher number of chronic illnesses were more likely to have an established healthcare provider.

Other factors, including age (B = 0.053, SE = 0.075, 95% CI = −0.094, 0.200, p = 0.477), gender (B = −0.108, SE = 0.062, 95% CI = −0.229, 0.013, p = 0.081), education level (B = 0.011, SE = 0.078, 95% CI = −0.142, 0.164, p = 0.890), and primary language (B = −0.072, SE = 0.072, 95% CI = −0.214, 0.069, p = 0.315), were not significant predictors of having a regular healthcare provider.

## 4. Discussion

Access to a regular provider was associated with telehealth use in this sample, while chronic illness burden was associated with having a regular provider. The adjusted direct association between illness burden and telehealth use was not statistically significant. This pattern is compatible with the proposed pathway, but the available results do not establish that provider status mediates the association or fully explains it.

This report extends Cobb et al. [32] by examining a different question within the same dataset. The shared participants, descriptive statistics, and correlations should therefore be understood as the basis for a continuation analysis. The potential contribution lies in examining regular provider access as an intermediate variable, rather than in presenting a new sample or independent confirmation of earlier findings.

The earlier study did not support perceived benefits or self-efficacy as mediators of telehealth use [32]. The current findings suggest that access to an established clinician deserves further study alongside these attitudes. A provider relationship may offer a route into remote care, although the present survey cannot determine whether that relationship preceded telehealth use or resulted partly from greater healthcare contact.

Education was not significantly associated with either regular provider status or telehealth use in the current model. Marginalization-Related Diminished Returns theory offers one possible context for interpreting weak associations between education and health-related resources [33, 34]. However, this analysis does not directly test that theory: it includes neither a White comparison group nor an estimate of differences in educational returns across racial groups. The null findings may also reflect limited variation, measurement, or statistical precision.

Women and English-speaking participants reported greater telehealth use in the adjusted model. Prior work has described gender differences in healthcare use [35-39] and language-related barriers to services [40-43]. In this setting, language support and accessible communication may be relevant to remote care. The associations do not identify the specific barriers encountered by participants, and explanations based on language access should be examined directly in future work.

A regular provider may help patients learn about remote appointments, arrange follow-up, and maintain contact with services. Earlier studies connect regular sources of care with access and patient-reported care experiences [47-50]. These possible explanations remain hypotheses here. Having a regular provider is a limited indicator of access and should not be treated as a direct measure of trust, relationship quality, or continuity over time.

Several limitations affect interpretation. The cross-sectional design does not establish the order of illness burden, provider access, and telehealth use, and greater use of care could itself increase the likelihood of reporting a regular provider. Nonrandom recruitment from four developments limits generalizability beyond the surveyed residents. Self-reported measures may be affected by recall and reporting differences. Insurance coverage, digital access, disability, provider availability, and other unmeasured factors could contribute to the observed associations. In addition, the reported paths alone do not demonstrate mediation; an estimated indirect effect with uncertainty is required. Because this is the same dataset analyzed by Cobb et al. [32], agreement between the two reports is not evidence of independent replication.

Future studies could follow residents over time to assess whether gaining a regular provider is followed by changes in telehealth use. Interviews could clarify how appointments are arranged and which language, technology, and service barriers residents encounter. Analyses that measure continuity directly, account for digital and insurance access, and estimate indirect effects would help assess the pathway proposed here. Broader recruitment would also help determine whether the pattern applies to other public housing communities.

## 5. Conclusions

In this continuation of Cobb et al. [32], chronic illness burden was associated with having a regular healthcare provider, and regular provider status was associated with telehealth use. These findings support further examination of provider access as a possible link between health needs and remote care. They do not establish a causal pathway or full mediation. Efforts to improve telehealth access may benefit from considering ongoing access to providers and language support alongside technology and patient attitudes.

## Figures and Tables

**Figure 1:**
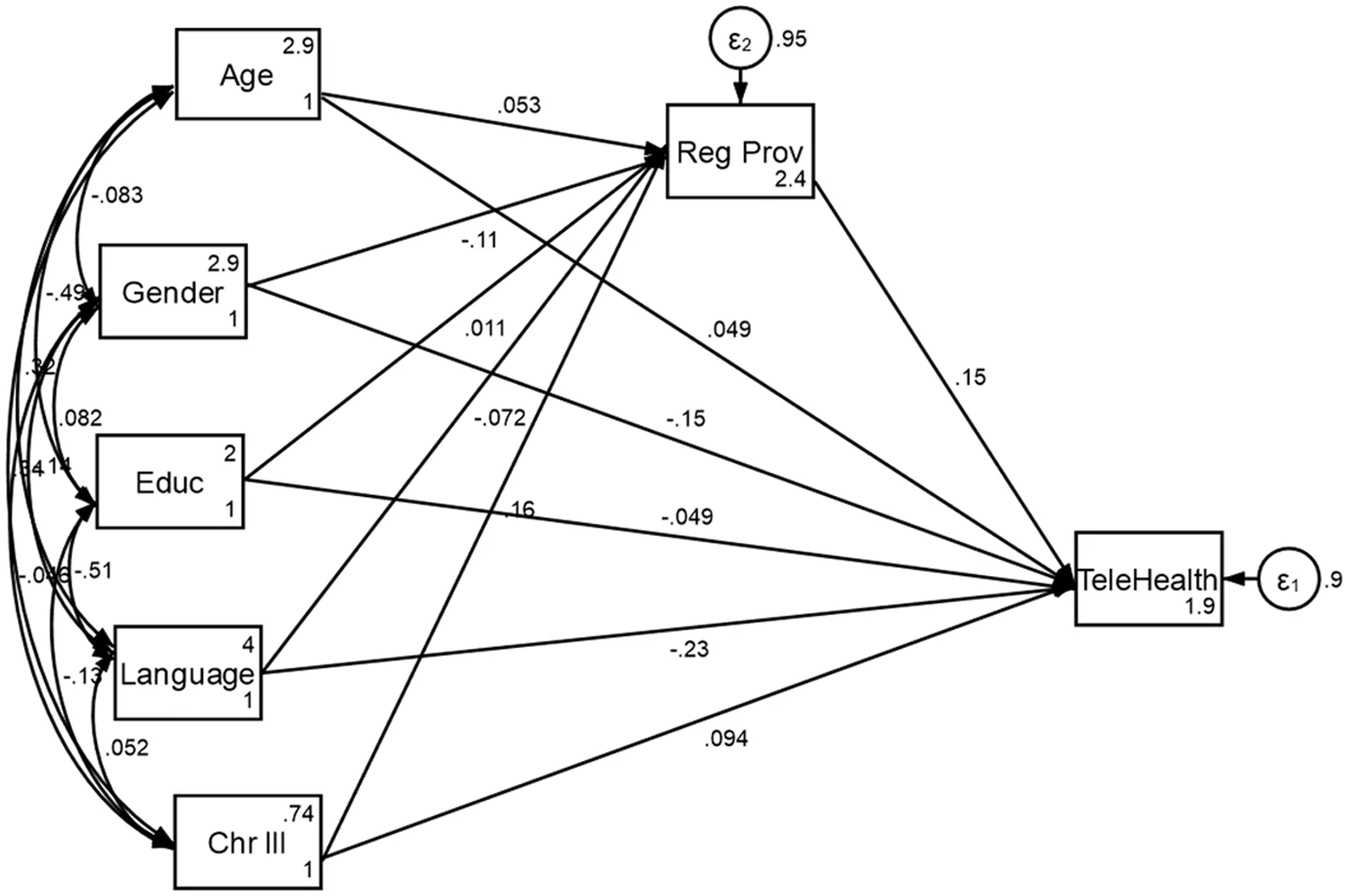
Proposed model relating chronic illness burden, regular provider status, and telehealth use. Chr Ill: number of chronic illnesses; Reg Prov: regular healthcare provider; Educ: educational attainment; Language: Spanish; Gender: male.

**Table 1:** Characteristics of the shared study sample (N = 252). Adapted from Cobb et al. [32]; retained values are unchanged, with measures outside the present analysis omitted.

Age (Yrs)	Mean	SD
	45	15
Number of Chronic Illnesses (0–8)	1	2
	N	%
Gender		
Male	64	25
Female	188	75
Education		
8th Grade or Less	99	39
Some High School, No diploma	56	22
Graduated from High/ GED equivalent	66	26
Associate Degree or Some College/Bachelor’s Degree	31	12
Language		
English	65	26
Spanish	187	74
Primary Care Provider		
No	45	18
Yes	207	82
Chronic Illnesses		
0	122	48
1	47	19
2	39	15
3 or more	44	18
Used Telehealth in the Past 12 Months		
0	133	53
1	50	20
2	37	15
3	19	7
4 or more	13	5

**Table 2:** Correlations among variables retained in the present analysis. Adapted from Cobb et al. [32]; all retained coefficients and significance markers are unchanged.

1. Age	1	2	3	4	5	6	7
	1.00						
2. Gender	−0.08	1.00					
3. Education	−0.49**	0.08	1.00				
4. Language	0.32**	−0.14*	−0.51**	1.00			
5. Number of Chronic Illnesses	0.34**	−0.05	−0.13*	0.05	1.00		
6. Primary Care Provider	0.09	−0.11	−0.01	−0.04	0.18**	1.00	
7. Telehealth Care Utilization	0.04	−0.14*	0.03	−0.15*	0.20**	0.20**	1.00

Note:

** Correlation is significant at the 0.01 level (two-tailed), and

* correlation is significant at the 0.05 level (two-tailed).

**Table 3:** Structural equation model examining regular provider status and telehealth use.

Telehealth use	B	SE	95%	CI	p
				
Regular Healthcare Provider	0.145	0.060	0.027	0.264	0.016
Age	0.049	0.073	−0.093	0.192	0.497
Gender	−0.153	0.060	−0.270	−0.035	0.011
Education	−0.049	0.076	−0.197	0.099	0.517
Spanish Language	−0.230	0.069	−0.365	−0.095	0.001
Chronic Illness (n)	0.094	0.064	−0.031	0.220	0.141
Intercept	1.871	0.505	0.881	2.861	< 0.001
Regular Healthcare Provider					
Age	0.053	0.075	−0.094	0.200	0.477
Gender	−0.108	0.062	−0.229	0.013	0.081
Education	0.011	0.078	−0.142	0.164	0.890
Spanish Language	−0.072	0.072	−0.214	0.069	0.315
Chronic Illness (n)	0.158	0.065	0.031	0.285	0.015
Intercept	2.448	0.510	1.449	3.447	< 0.001
Mean					
Age	2.949	0.146	2.663	3.234	< 0.001
Gender	2.881	0.143	2.601	3.161	< 0.001
Education	1.987	0.109	1.774	2.199	< 0.001
Spanish Language	3.982	0.188	3.613	4.351	< 0.001
Chronic Illness (n)	0.744	0.071	0.605	0.884	< 0.001

## Data Availability

The original contributions presented in this study are included in this article. Further inquiries can be directed to the corresponding authors.
